# Correlation between vascular endothelial growth factor A gene polymorphisms and tendon and ligament injury risk: a systematic review and meta-analysis

**DOI:** 10.1186/s13018-024-04589-z

**Published:** 2024-02-06

**Authors:** Xi-yong Li, Yun-lu Wang, Su Yang, Chang-sheng Liao, Song-feng Li, Peng-fei Han

**Affiliations:** 1 Department of Orthopaedics, Heping Hospital Affiliated to Changzhi Medical College, 110 South Yan’an Road, Changzhi, 046000 People’s Republic of China; 2 Graduate School, Changzhi Medical College, Changzhi, People’s Republic of China

**Keywords:** *VEGFA*, Gene polymorphisms, Tendon injury, Ligament injury, Meta

## Abstract

**Background:**

Relevant evidence suggests that angiogenic factors contribute significantly to fibril matrix reconstruction following physical injuries to tendon ligaments. Vascular endothelial growth factor A (*VEGFA*), with its potent angiogenic effect, has been studied extensively, and its functional polymorphisms, including rs699947, rs1570360, and rs2010963, have been the focus of numerous investigations. Some scholars have explored the association between gene polymorphisms in the *VEGFA* and the risk of tendon ligament injury, but the findings are not entirely consistent.

**Objectives:**

The purpose of this study was to investigate the association between rs699947, rs1570360, and rs2010963 gene polymorphisms in *VEGFA* and the risk of tendon and ligament injuries.

**Methods:**

After including articles about the association of *VEGFA* rs699947, rs1570360, and rs2010963 polymorphisms with tendon and ligament injuries according to the search strategy, we assessed their quality and conducted meta-analyses to examine the link between these polymorphisms and the risk of tendon and ligament injuries using odds ratios and 95% confidence intervals.

**Results:**

Of 86 related articles, six were included in the meta-analysis. Some of these suggest an association between *VEGFA* rs2010963 and the risk of tendon and ligament injury in the population, with the specific C allele being one of the adverse factors for knee injury. Some studies suggest that *VEGFA* rs699947 and *VEGFA* rs1570360 single-nucleotide polymorphisms are associated with anterior cruciate ligament rupture. The risk of non-contact anterior cruciate ligament rupture is nearly doubled in individuals with the rs699947 CC genotype compared to the control group. Our analysis did not find any significant relationship between *VEGFA* gene polymorphisms (rs699947, rs1570360, and rs2010963) and the chance of tendon and ligament injury without consideration of race. However, the European population reveals that the CC genotype of *VEGFA* rs699947 can result in a greater risk of tendon and ligament injury, whereas the AG genotype for rs1570360 provides some protection. Additionally, rs2010963 was significantly associated with tendon and ligament injury; individuals with the C allele and the CC genotype had higher risk. False-positive report probability confirmed the high credibility of our results.

**Conclusion:**

Overall, this study found no significant association between *VEGFA* rs699947, rs1570360, and rs2010963 polymorphisms and the risk of tendon ligament injury. However, in subgroup analysis, some genotypes of *VEGFA* rs699947, rs1570360, and rs2010963 were found to increase the risk of tendon ligament injury in European populations.

## Introduction

As sports medicine continues to evolve, tendon and ligament injuries are receiving more and more attention. There are approximately 16.5 million reported cases of tendon and ligament injuries in the USA each year, which not only reduces the quality of life of patients, but also increases the socioeconomic burden [1]. Tendons and ligaments aid in the transmission of muscle strength while upholding joint stability, necessitating them to endure tremendous strain in daily life responsibilities, particularly during physical activity [2]. According to statistics, a professional soccer player sustains two injuries per season, with a higher likelihood of lower limb injuries. Injuries, such as Achilles tendon and anterior cruciate ligament injuries, can critically impact their athletic careers and daily lives [3, 4]. ACL injuries reportedly account for 30% of all knee injuries in high school athletes, with a higher incidence in females than males [5]. Although materials, such as autologous tendons or artificial tendons, can be used to reconstruct the stability of ligaments, a significant number of patients find it difficult to fully recover to their preoperative physical condition. Additionally, ligament reconstruction is a significant risk factor for postoperative ligament re-rupture [6]. And more than half of ACL injuries are non-contact injuries [7]. Achilles tendon disorder is a frequent injury among athletes. The majority of patients occur around the age of 50, according to the study [8]. Meanwhile, the Danish study revealed that the peak sports injury is in September due to concentrated large-scale activities during the summer, implying that aging and overexertion remain critical factors in exacerbating tendon ligament injury risks [8]. While exercise does increase the load on the Achilles tendon, studies suggest that 65% of Achilles tendinopathy cases are not related to exercise [9, 10]. However, it is important to note that body mass index (BMI) is not a consistent risk factor for tendon and ligament injury. Research has shown that individuals with a high BMI have a lower likelihood of recurrent ACL injury, while those with a low BMI are at a heightened risk of secondary tendon and ligament injury [11]. The study discovered that women face a higher susceptibility to ACL injury compared to men. This is attributed to women having a greater degree of knee valgus range of motion upon landing [12]. Similarly, Fares et al. found that the excessively large posterior tibial slope (PTS) is an important risk factor for increased ACL injury [13]. Tendon ligament injuries are influenced by age, sex, movement style, and location of training. Therefore, preventing these injuries is the key to treatment [14].

Research has shown that the formation of new blood vessels can greatly affect tendons and ligaments, particularly during injury repair. As a result, promoting the formation of blood vessels via biological and mechanical stimulation can hasten recovery [15]. *VEGFA* is a prominent angiogenic agent situated on chromosome 6's short arm. It is made up of an 8-exon and 7-intron coding area of 14-kb and is exceedingly polymorphic [16]. The most prevalent type of genetic variation is the single-nucleotide polymorphisms (SNPs) that arise from the substitution of just a single nucleotide. SNPs or mutations may relate to the predisposition to diseases, the development of diseases, and the effectiveness of targeted medications [17, 18]. The *VEGFA* promoter contains several typical single-nucleotide polymorphisms (SNPs) that functionally regulate *VEGFA* expression. These SNPs include −2578C/A (rs699947), −1154G/A (rs1570360), −634C/G (rs2010963), and +936C/T (rs3025039). Among them, rs699947 (C/A), rs1570360 (G/A), and rs2010963 (G/C) are the most commonly studied SNPs associated with angiogenesis, located at the −2578, −1154, and −634 translation start sites in the promoter region [16, 19]. *VEGFA*, the most angiogenic subtype of the five VEGF subtypes, plays a critical role in regulating the extracellular matrix of tendons and ligaments [20]. Therefore, several studies indicate that genetic variations in *VEGFA* may be connected to the likelihood of sustaining tendon or ligament injuries [21]. Research has found that the *SP1* TT polymorphism present in the *COLIA1* genotype is linked to an increased risk of cruciate ligament injury [22]. Additionally, the polymorphism of the *COL5A1* gene is associated with the risk of Achilles tendon and quadriceps tendon injuries, as well as anterior cruciate ligament tears [23, 24]. Furthermore, *COL5A1* can interact with the *MMP3* gene, which encodes matrix metalloproteinase, thereby heightening the probability of Achilles tendinopathy occurrence [25, 26]. This study compares the relationship between rs699947, rs1570360, and rs2010963 gene polymorphisms and tendon ligament injury risk in *VEGFA* for the first time through meta-analysis, hoping to provide some help for the prevention of related diseases and further personalized treatment.

## Materials and methods

### Search strategy

The current meta-analysis was conducted following the Preferred Reporting Items for Systematic Reviews and Meta-Analyses (PRISMA) checklist. We have registered it prospectively at PROSPERO(CRD42023460376). Relevant literature in the databases, including EMBASE, PubMed Central, Web of Science, Cochrane Library, CNKI, and Wanfang Data Knowledge Service Platform, were searched to analyze the relationship between *VEGFA* gene polymorphisms and tendon ligament injury. The search strategy was ("vascular endothelial growth factor" or "*VEGFA*" or "vascular permeability factor" or "VPF") and ("polymorphism" or "variant" or "variation" or "mutation" or "SNP" or "genome-wide association study" or "genetic association study" or "genotype" or "allele") and ("tendon" or "ligament" or "Achilles tendon" or "Anterior cruciate ligament" or "Patellar tendon" or "Elbow tendons"). The search deadline was January 2023.

### Inclusion and exclusion criteria

Inclusion Criteria: (1) case–control and cohort studies; (2) correlation between *VEGFA* rs699947, rs1570360, and rs2010963 polymorphisms and tendon and ligament injuries; (3) detailed control and case group genotype data or their OR with 95% CI. Exclusion criteria: (1) reviews, systematic reviews, case reports, letters, and republished studies; (2) non-case–control studies; (3) literature with incomplete genotypes and irrelevant literature.

### Data extraction and quality evaluation

Two independent researchers extracted data separately using a strict standard protocol. When disagreements arose, they were resolved through discussion or jointly evaluated with a more senior researcher until a consensus was reached. The extracted information includes the first author, publication year, study country, ethnicity, case and control source, participant sex, number of cases and controls, number of distributed genotypes, diagnostic criteria for tendon ligament injury, and investigators' conclusions. The subject selection, inter-group comparability, and outcome measures for all included studies were evaluated using the Newcastle–Ottawa–Scale (NOS). The higher the total score, the higher the quality of the study. The NOS score was divided into three grades: low, medium, and high quality, namely, < 5, 5–7, and 8–9 points.

### Statistical methods

Meta-analysis of data extracted from the included studies was conducted using Stata 17.0 software. The strength of association was assessed using ORs with respective 95% CIs and was considered statistically significant when the *P* < 0.05. The study compared five genetic models: allele model, additive model, dominant model, recessive model, and over-dominant model. Additionally, a subgroup analysis was conducted for further investigation. Heterogeneity was evaluated using chi-square-based *Q* and *I*^2^ values. *P* > 0.10 or *I*^2^ < 50% indicated no noteworthy heterogeneity among the included studies, which necessitated the use of a fixed-effect model. When significant heterogeneity was present, a random-effects model was employed. Two sensitivity analyses were performed by (1) removing one of the included studies and (2) eliminating studies that did not comply with Hardy–Weinberg equilibrium (HWE). Egger testing and funnel plots were utilized to identify publication bias, while false-positive report probability (FPRP) was employed to assess confidence in all positive outcomes.

## Results

### Basic characteristics of the included literature

**Fig. 1:**
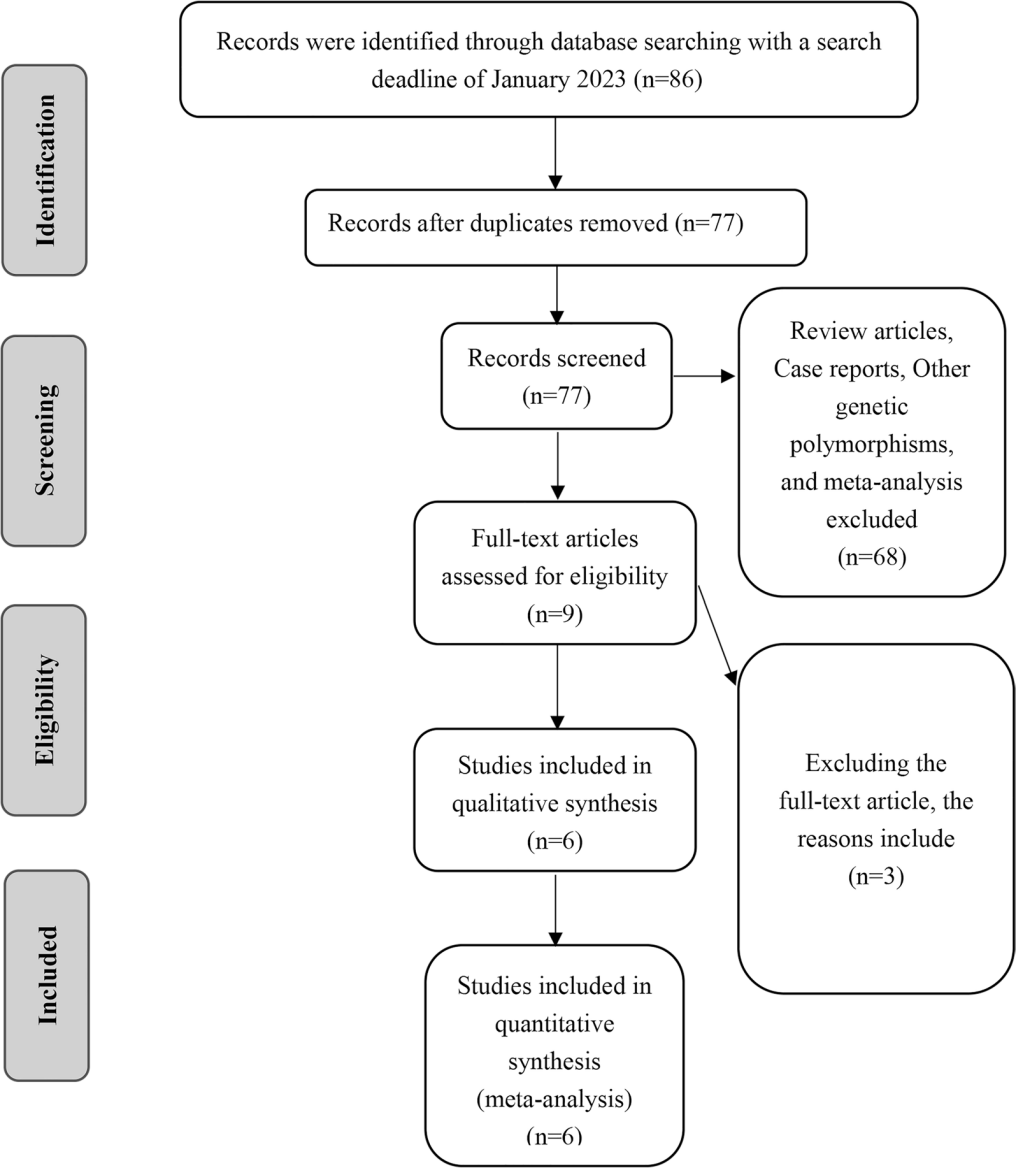
Flow diagram of literature searching

Eighty-six relevant papers were retrieved from major databases via strict implementation of the inclusion criteria. After excluding publications that were not case–control studies, duplicate publications, and other irrelevant literature, nine relevant papers were preliminarily screened; six papers (with 1061 individuals in the case group and 986 individuals in the control group) were finally included after careful reading of the full text. Six articles investigated rs699947, and four articles evaluated rs1570360 and rs2010963. The literature screening process and results are shown in Fig. 1, and the basic characteristics of the included studies are shown in Table 1 [19, 27–31]. The mode of injury and occupational status within the case group are presented in Table 2, and Tables 3, 4 and 5 show the detailed gene frequencies of *VEGFA* rs699947, rs1570360, and rs2010963 polymorphisms.

**Table 1:** Main characteristics and quality score of studies included

References	Country	Ethnicity	Gender (F/M)	Cases	Controls								NOS score
				*N*	Age^a^	Of cite	Diagnosis	Matching	*N*	Age^a^	HWE	Healthy	
Cięszczyk et al. [27]	Poland	Caucasian	115/257	229	26 ± 4	ACL	Surgery	Age and Sex	143	24.2 ± 4.1	HWE	Yes	7
Shukla et al. [28]	India–United Kingdom	Indo-Pakistani	32/134	90	26.6 ± 6.2	ACL	Radiology or Surgery	Age and Sex	76	62.2 ± 5.7	HWE	Yes	7
Rahim et al.[29]	South Africa	Caucasian	SA 78/150	108	42.9 ± 13.6	Achilles Tendon	WHO	Age and Sex	120	37.3 ± 10.4	HWE	Yes	6
	United Kingdom		UK 84/133	87	45.2 ± 14.4				130	41.6 ± 11.6	HWE	Yes	
Rahim et al.[30]	South Africa	Colored	36/162	98	24.5 ± 7.5	ACL	WHO	Age and Sex	100	27.4 ± 6.7	HWE	Yes	5
Rahim et al.[31]	South Africa	Caucasian	151/310	227	26.8 ± 11.0	ACL	WHO	Sex	234	29.3 ± 11.3	HWE	Yes	6
Lulińska-Kuklik et al. [19]	Poland	Caucasian	149/263	222	F 25 ± 4	ACL	Surgery	Sex	190	F 29 ± 2	HWE	Yes	7
					M 26 ± 4					M 25 ± 2			

*F* female, *M* male, *NA* not available, *ACL* anterior cruciate ligament, *WHO* World Health Organization diagnostic criteria, *HWE* Hardy–Weinberg equilibrium, *a* mean ± SD, *NA* not available

**Table 2:** Profession and mode of injury in the case groups of included studies

Group	References					
	Cięszczyk et al. [27]	Shukla et al. [28]	Rahim et al. [29]	Rahim et al. [30]	Rahim et al. [31]	Lulińska-Kuklik et al. [19]
Profession						
Case	Football player	Athletes	Patients with achilles tendinopathy	Healthy people	Healthy people	Football player
Control	Football player	Athletes	Healthy people	Healthy people	Healthy people	Healthy people
Mode of injury of case group						
Contact	0	62	NA	47	101	0
Non-contact	229	28	NA	51	126	222

*NA* not available

**Table 3:** Genotype frequencies of *VEGFA* rs699947 polymorphism in studies included in this meta-analysis

References	Country	Ethnicity	HWE		Number of samples			Genotypes of cases			Alleles of cases		Minor allele frequency	Genotypes of controls			Controls’ alleles		Minor allele frequency
			*χ*^2^	*P*	Cases	Controls	Total	A/A	A/C	C/C	A	C		A/A	A/C	C/C	A	C	
Cięszczyk et al. [27]	South Africa	Caucasian	1.5498	0.2131	143	229	372	33	84	26	150	136	0.4755	55	114	60	224	234	0.4890
Shukla et al. [28]	India	Indo-Pakistani	0.0112	0.9156	90	76	166	19	51	20	89	91	0.4944	13	30	33	56	96	0.3684
Rahim et al.[29]	South Africa	Caucasian	0.5679	0.4510	166	229	395	39	92	35	170	162	0.4879	62	98	69	222	236	0.4847
Rahim et al.[30]	South Africa	Colored	0.4862	0.4856	96	95	191	12	48	36	72	120	0.3750	10	44	41	64	126	0.3368
Rahim et al.[31]	South Africa	Caucasian	0.3763	0.5395	223	226	449	52	106	65	210	236	0.4708	57	125	44	239	213	0.4712
Lulińska-Kuklik et al. [19]	Poland	Caucasian	3.6690	0.05543	222	190	412	39	121	62	199	245	0.4481	25	99	66	149	231	0.3921

*HWE* Hardy–Weinberg equilibrium, *χ*^2^ Chi-square, *NA* not available

**Table 4:** Genotype frequencies of *VEGFA* rs1570360 polymorphism in studies included in this meta-analysis

References	Country	Ethnicity	HWE		Number of samples			Genotypes of cases			Alleles of cases		Minor allele frequency	Genotypes of controls			Controls’ alleles		Minor allele frequency
			*χ*^2^	*P*	Cases	Controls	Total	A/A	A/G	G/G	A	G		A/A	A/G	G/G	A	G	
Rahim et al.[29]	South Africa	Caucasian	2.3295	0.1269	160	216	376	20	63	77	103	217	0.3218	32	94	90	158	274	0.3657
Rahim et al.[30]	South Africa	Colored	43.5068	0.0001	95	97	192	19	19	57	57	133	0.3000	17	23	57	57	137	0.2938
Rahim et al.[31]	South Africa	Caucasian	0.6668	0.4141	224	212	436	21	108	95	150	298	0.3348	28	75	109	131	293	0.3089
Lulińska-Kuklik et al. [19]	Poland	Caucasian	1.6655	0.1968	312	190	502	19	95	198	133	491	0.2131	11	69	110	91	289	0.2394

*HWE* Hardy–Weinberg equilibrium, *χ*^2^ Chi-square, *NA* not available

**Table 5:** Genotype frequencies of *VEGFA* rs2010963 polymorphism in studies included in this meta-analysis

References	Country	Ethnicity	HWE		Number of samples			Genotypes of cases			Alleles of cases		Minor allele frequency	Genotypes of controls			Controls’ alleles		Minor allele frequency
			*χ*^2^	*P*	Cases	Controls	Total	C/C	C/G	G/G	C	G		C/C	C/G	G/G	C	G	
Rahim et al.[29]	South Africa	Caucasian	0.0228	0.8797	167	232	399	17	77	73	111	223	0.3323	26	101	105	153	311	0.3297
Rahim et al.[30]	South Africa	Colored	0.3221	0.5703	99	93	192	7	38	54	52	146	0.2626	7	32	54	46	140	0.2473
Rahim et al.[31]	South Africa	Caucasian	0.0774	0.7808	227	226	453	24	99	104	147	307	0.3237	29	101	96	159	293	0.3517
Lulińska-Kuklik et al. [19]	Poland	Caucasian	0.0106	0.9176	222	190	412	52	107	63	211	233	0.4752	27	97	66	151	229	0.3973

*HWE* Hardy–Weinberg equilibrium, *χ*^2^ Chi-square, *NA* not available

### Results of meta-analyses

**Fig. 2:**
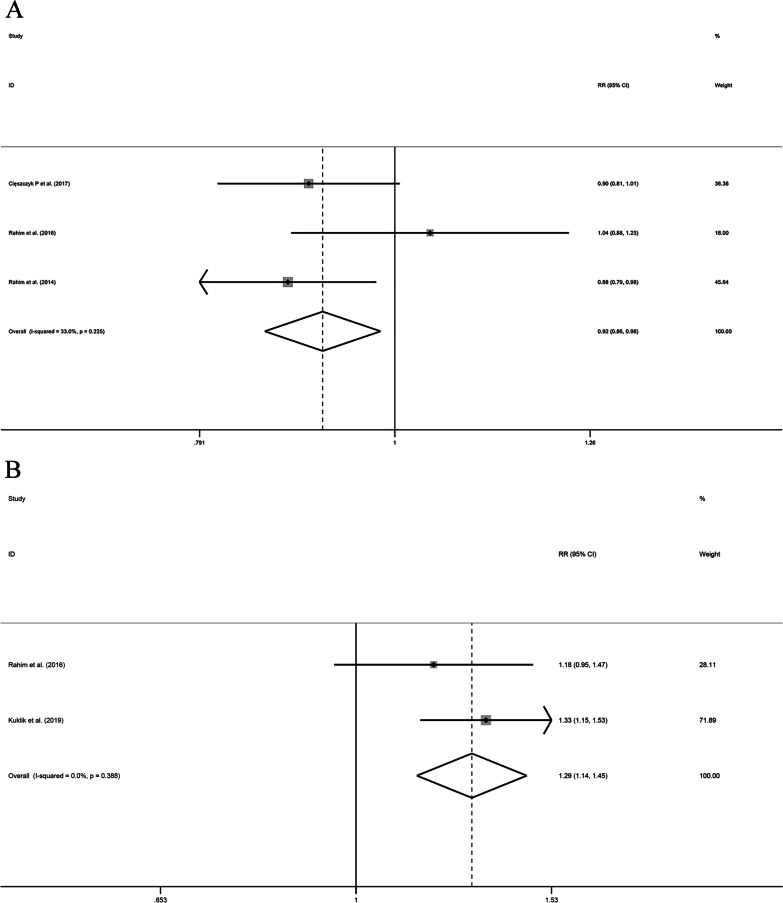

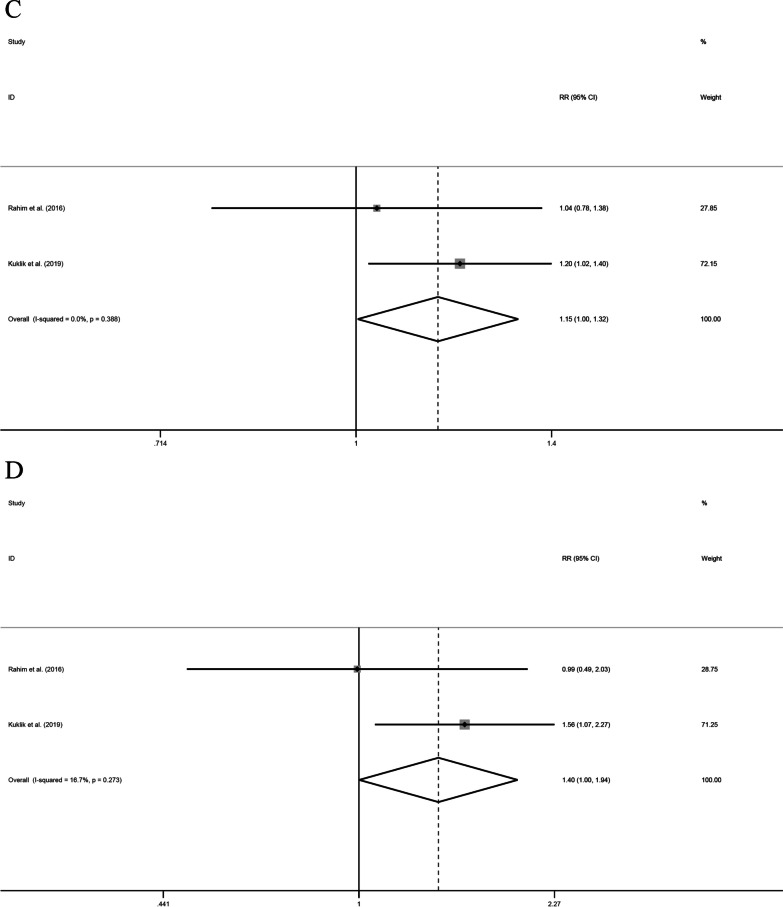
Forest plots of all selected studies on the association between *VEGFA* polymorphism and the risk of tendon ligament injury in Europeans (**A** rs699947 dominant model, **B** rs1570360 over-dominant model, **C** rs2010963 allele model, and **D** rs2010963 additive model), *CI* confidence interval, *RR* risk ratio

Six studies examined the link between *VEGFA* rs69947 gene polymorphisms and the susceptibility to tendon and ligament injuries. However, none of the five genotypes displayed a significant association. Because four of the articles involved European populations, we performed subgroup analyses. There were no significant differences in the allele, additive, over-dominant, or recessive models. However, in the dominant model, heterogeneity decreased from 66.9 to 33% after removing the study by Lulińska-Kuklik et al. [19]; a fixed-effect model was then used to further analyze this finding (OR 0.92, 95% CI 0.86–0.98, *P* = 0.015). These results indicate that the *VEGFA* rs699947 AA and AC genotypes are associated with a reduced risk of tendon and ligament injury in European populations (Fig. 2).

Four papers investigated gene polymorphisms in *VEGFA* rs1570360, and there were no significant differences in all gene models. In a subgroup analysis of the European population, the over-dominant model showed a directional change in heterogeneity after removing the study by Rahim et al. [31]. When this study was excluded, both the AA and GG genotypes in *VEGFA* rs1570360 increased the risk of tendon and ligament injury in the European population (OR 1.29, 95% CI 1.14–1.45, *P* < 0.001, Fig. 2). Similarly, four studies investigated the associations between *VEGFA* rs2010963 gene polymorphisms and tendon and ligament injury risk. There were no significant differences in any of the models. In European populations, the allele and additive gene models had reduced heterogeneity (from 56.2 to 0% and 57.4 to 16.7%, respectively) after removing data from each of two studies by Rahim et al. [29] and [31], respectively). In the European population, the G allele in *VEGFA* rs1570360 had a protective effect against tendon and ligament injury (OR 1.15, 95% CI 1.00–1.32, *P* = 0.045, Fig. 2), and the GG genotype was associated with a lower risk of tendon and ligament injury than the CC genotype (OR 1.40, 95% CI 1.00–1.94, *P* = 0.049). These data are shown in detail in Table 6 and 7.

**Table 6:** Pooled estimates of association of *VEGFA* rs699947, rs1570360, and rs2010963 polymorphisms and the risk of tendon injury

Genetic model	Test of association	Tests for heterogeneity		Egger’s test	
	OR (95% CI)	*P*	*P*_h_	*I*^2^ (%)	*P*_E_
*VEGFA* rs699947					
A versus C	1.08 (0.88–1.33)	0.455	0.029	59.90	0.184
AA+AC versus CC	1.16 (0.74–1.80)	0.514	0.001	78.30	0.423
AA versus CC	1.13 (0.75–1.71)	0.550	0.048	55.30	0.232
AA versus AC+CC	1.03 (0.83–1.28)	0.792	0.706	0	0.189
AA+CC versus AC	0.90 (0.65–1.26)	0.556	0.005	69.90	0.330
*VEGFA* rs1570360					
A versus G	0.95 (0.81–1.11)	0.511	0.431	0	0.943
AA+AG versus GG	0.96 (0.70–1.31)	0.791	0.088	54.1	0.905
AA versus GG	0.88 (0.63–1.25)	0.484	0.857	0	0.212
AA versus AG+GG	0.88 (0.63–1.22)	0.426	0.661	0	0.117
AA+GG versus AG	1.53 (0.90–2.61)	0.115	0.001	84.4	0.520
*VEGFA* rs2010963					
C versus G	1.07 (0.92–1.25)	0.364	0.163	41.5	0.997
CC+CG versus GG	1.07 (0.87–1.32)	0.523	0.502	0	0.555
CC versus GG	1.16 (0.83–1.62)	0.393	0.124	47.9	0.744
CC versus CG+GG	1.15 (0.84–1.57)	0.371	0.140	45.3	0.580
CC+GG versus CG	1.00 (0.81–1.23)	0.978	0.808	0	0.324

rs699947: allele model: A versus C, dominant model: AA + AC versus CC, additive model: AA versus CC, recessive model: AA versus AC + CC, over-dominant model: AA + CC versus AC

rs1570360: allele model: A versus G, dominant model: AA + AG versus GG, additive model: AA versus GG, recessive model: AA versus AG + GG, over-dominant model: AA + GG versus AG

rs2010963: allele model: C versus G, dominant model: CC + CG versus GG, additive model: CC versus GG, recessive model: CC versus CG + GG, over-dominant model: CC + GG versus CG

**Table 7:** Pooled estimates of the association of *VEGFA* rs699947, rs1570360, and rs2010963 polymorphisms with tendon injury risk in Europeans

Genetic model	Test of association	Tests for heterogeneity		Egger’s test	
	OR (95% CI)	*P*	*P*_h_	*I*^2^ (%)	*P*_E_
*VEGFA* rs699947					
A versus C	0.98 (0.88–1.10)	0.758	0.109	50.50	0.356
AA + AC versus CC	0.92 (0.86–0.98)	0.015	0.225	33.00	0.075
AA versus CC	0.95 (0.81–1.12)	0.534	0.112	50.00	0.059
AA versus AC+CC	0.99 (0.83–1.17)	0.877	0.377	3.1	0.022
AA+CC versus AC	1.01 (0.83–1.24)	0.910	0.010	73.7	0.787
*VEGFA* rs1570360					
A versus G	0.95 (0.84–1.09)	0.467	0.186	40.5	0.133
AA+AG versus GG	1.01 (0.82–1.24)	0.953	0.085	59.5	0.555
AA versus GG	0.86 (0.61–1.21)	0.381	0.844	0	0.875
AA versus AG+GG	0.84 (0.59–1.19)	0.326	0.678	0	0.006
AA+GG versus AG	1.29(1.14–1.45)	0.001	0.388	0	Na
*VEGFA* rs2010963					
C versus G	1.15 (1.00–1.32)	0.045	0.388	0	Na
CC+CG versus GG	1.03 (0.93–1.13)	0.583	0.351	4.4	0.877
CC versus GG	1.40 (1.00–1.94)	0.049	0.273	16.7	Na
CC versus CG+GG	1.12 (0.69–1.82)	0.642	0.094	57.6	0.568
CC+GG versus CG	1.01 (0.91–1.13)	0.799	0.702	0	0.544

rs699947: allele model: A versus C, dominant model: AA+AC versus CC, additive model: AA versus CC, recessive model: AA versus AC+CC, over-dominant model: AA+CC versus AC

rs1570360: allele model: A versus G, dominant model: AA+AG versus GG, additive model: AA versus GG, recessive model: AA versus AG+GG, over-dominant model: AA+GG versus AG

rs2010963: allele model: C versus G, dominant model: CC+CG versus GG, additive model: CC versus GG, recessive model: CC versus CG+GG, over-dominant model: CC+GG versus CG

Statistical significance values are shown in bold, NA: not available

### Publication bias and sensitivity analyses

**Fig. 3:**
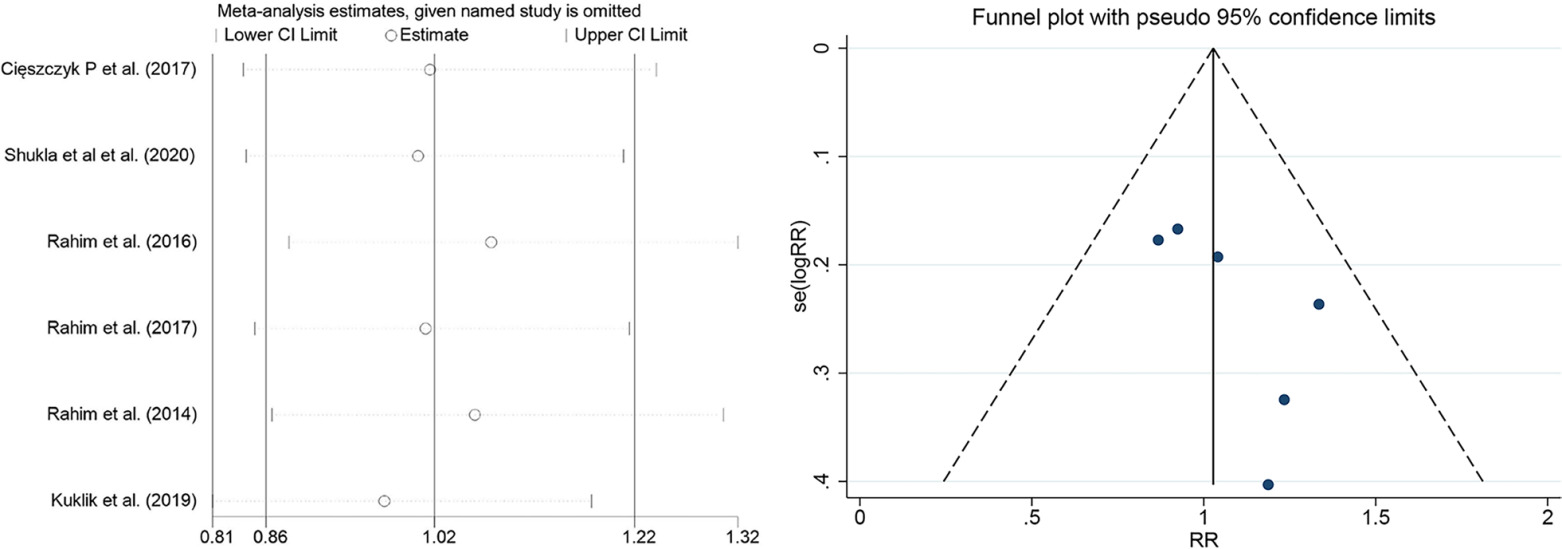
Sensitivity analysis and publication bias funnel plot of the *VEGFA* polymorphisms and risk of tendon ligament injury

**Fig. 4:**
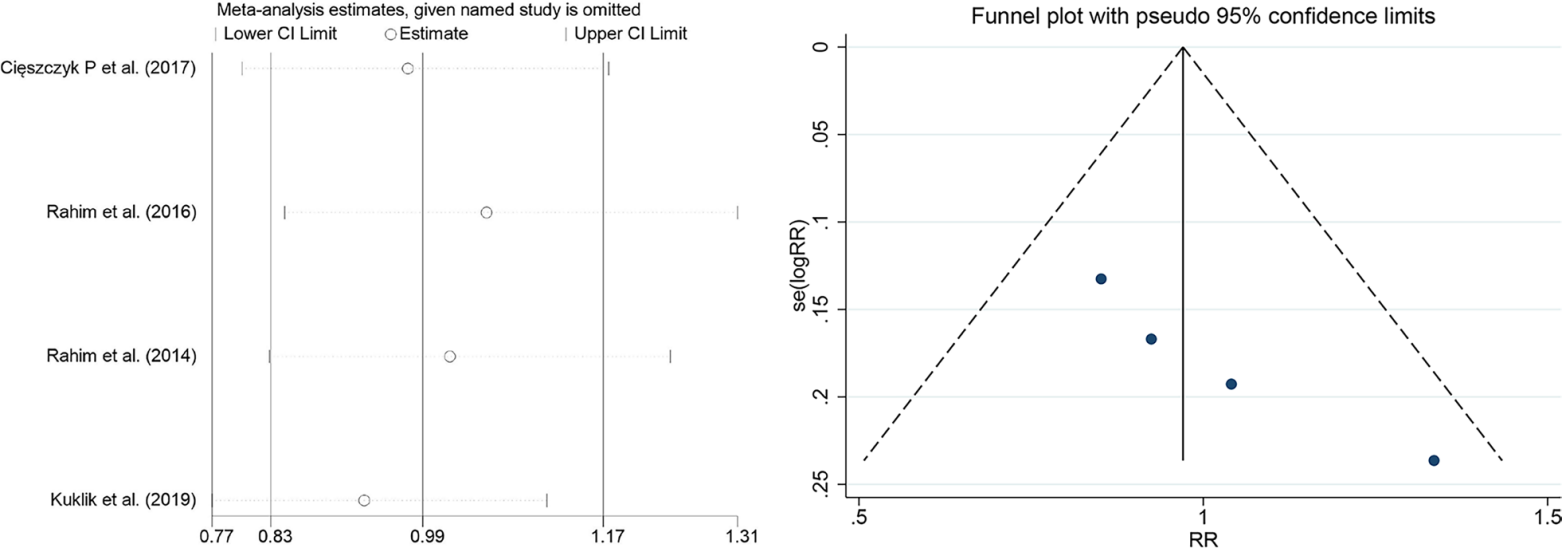
Sensitivity analysis and publication bias funnel plot of the *VEGFA* polymorphisms and risk of tendon ligament injury in Europeans

The meta-analysis found significant heterogeneity between individual studies, possibly due to factors such as race, sex, and age. No subgroup analyses were performed as gender and age were not grouped in the included studies. In the subgroup analysis of races, we found that after the European population excluded Kulik et al. (2019) in the dominant model of rs699947, Rahim et al. [31] in the over-dominant model of rs1570360, and deleted Rahim et al. [29] and Rahim et al. [31] in the allele model and additive model of rs2010963, respectively, the *I*^2^ value changed from > 50 to < 50%, so there were considered as a source of heterogeneity and removed. The studies that remained showed no change in heterogeneity when the included papers were excluded individually. To assess publication bias, both the Egger test and funnel plot were used. The results were relatively stable and there was no clear publication bias, as shown in the funnel chart (Figs. 3 and 4) and Egger detailed data (Table 6 and 7).

### Confidence test for positive results

All positive results are evaluated for FPRP values under various prior probability conditions by OR and 95% CI in order to determine whether they are truly associated with the risk of tendon ligament injury. A FPRP value < 0.2 is considered to indicate high confidence in the results. The confidence tests conducted in this meta-analysis found that the statistically significant positive results were reliable (Table 8).

**Table 8:** FPRP values for meta-analysis results

Positive result	Subgroup	Genetic model	OR (95% CI)	*I*^2^ (%)	*P*	Power	Prior probability				
							0.25	0.1	0.01	0.001	0.0001
*VEGFA* rs699947	Europe	AA+AC versus CC	0.92 (0.86–0.98)	33	0.015	1.00	**0.028**	**0.080**	0.490	0.906	0.990
*VEGFA* rs1570360	Europe	AA+GG versus AG	1.29 (1.14–1.45)	0	< 0.001	0.994	** < 0.001**	** < 0.001**	**0.002**	**0.019**	**0.165**
*VEGFA* rs2010963	Europe	C versus G	1.15 (1.00–1.32)	0	0.045	1.00	**0.123**	0.297	0.823	0.979	0.998
*VEGFA* rs2010963	Europe	CC versus GG	1.40 (1.00–1.94)	16.7	0.049	0.661	**0.164**	0.371	0.866	0.985	0.998

Statistical significance values are shown in bold

### Haplotypes analysis

VEGF has several commonly studied SNPs (−2578 C/A, −460T/C, −1154 G/A, +405 G/C, and +936 C/T) that may be associated with susceptibility to certain diseases. For instance, Xia Han et al. conducted haplotype analysis and discovered that T–C–T, C–C–C, and C–G–C haplotypes were all genetic susceptibility factors for coronary heart disease (OR: 2.43, 2.77, and 2.33) based on VEGF SNP (−460T/C, −634G/C, and 936C/T) [32]. Similarly, Eun-Ju Ko et al. found that VEGF −1154G>A, −1498T>C, +936C>T, +1451C>T, +1612G>A, +1725G>A haplotypes G–T–T–C–G, G–C–C–A–A, and A–T–C–G–G were strongly correlated with coronary artery disease sensitivity in their populations [33]. Haplotype analysis between VEGF −2578 C/A, −460T/C, −1154 G/A, and +405 G/C SNP found that haplotype C–T–G–G had a higher risk of endometriosis than haplotype C–C–G–G and A–T–G–G [34]. The above studies suggest that the SNPs of VEGF and the haploids that are composed between them do affect the susceptibility of some parts of this population to certain diseases. In this included study, Rahim et al. [29] found that haploid A–G–G of *VEGFA* (−2578C/A, −1154G/A, −634C/G) was positively correlated with Achilles tendinopathy. Lulińska-Kuklik et al. [19] believed that haploid C–G–C of *VEGFA* (−2578C/A, −1154G/A, −634C/G) increases the risk of ACL injury, while haploid C–G–G has the effect of protecting the ACL.

## Discussion

Tendon and ligament injuries commonly arise during physical activity. Incomplete statistics suggest that the likelihood of Achilles tendon injuries in athletes is approximately five times higher than that in the general population [10]. When tendon ligaments are damaged, it often leads to pain and discomfort, which can significantly impact one's quality of life. Anterior cruciate ligament injuries are mainly non-contact injuries in sports, with offensive running being the most prevalent cause [35]. Some studies have found that a greater ankle flexion angle does not protect the ACL because the heel does not make full contact with the ground, causing the calf muscles to be unable to fully absorb the reaction force from the ground, increasing stress on the knee joint [36]. Achilles tendinopathy is caused by the reduction of negative pressure tolerance and continuous overload of the Achilles tendon, which leads to degeneration and failure of healing of the Achilles tendon. Its characteristics mainly include local or diffuse increase in thickness, loss of normal collagen, and loss of normal tissue [37]. Although the two are not identical in terms of pathology, there may be certain similarities in the underlying causes. Therefore, it is important to identify the cause of tendon ligament injury and prevent its occurrence. There are many causes of tendon ligament injury, and in addition to external factors such as body weight and exercise, genetic contributions are also receiving more and more attention [38]. Studies have found that VEGF rises to preoperative levels up to 16 times after ACL injury [39, 40]. As one of the most potent subtypes, *VEGFA* could potentially assist in the clinical prevention of tendon ligament injuries by investigating the link between its gene polymorphism and the likelihood of tendon ligament injury. Through statistical analysis, it was found that there was no significant statistical difference between *VEGFA* rs699947, rs1570360, and rs2010963 gene polymorphisms and the risk of tendon ligament injury without distinguishing populations, and the probability of damage in each genotype was basically the same. In the subgroup analysis of the European population, we found that the population with AA and AC genotypes in the dominant model of *VEGFA* rs699947 had a lower probability of tendon ligament injury than the population with CC genotype, and the difference was statistically significant. Similarly, in the *VEGFA* rs1570360 over-dominant model, the AG genotype had a statistically significant difference in protecting tendon ligament injury in the European population compared with other genotypes. Studies have found that the rs1570630 GG genotype is linked to elevated expression of VEGFA. However, the overexpression of VEGFA may also decrease the biomechanical strength of tendons [20]. Additionally, individuals with the rs1570630 GG genotype tend to be heavier than those with other genotypes [41], which further raises their risk of tendon and ligament injuries. In the allele model of *VEGFA* rs2010963, G gene has the effect of reducing tendon ligament injury, and people with GG genotype in the additive model also have a lower risk of tendon ligament injury.

This meta-analysis has the following advantages: (1) the relationship between *VEGFA* rs699947, rs1570360, and rs2010963 gene polymorphisms and the risk of tendon ligament injury was studied for the first time, which was the most innovative in this study; (2) all included studies were assessed and scored in detail; (3) HWE calculations are performed on genotype frequencies to ensure that the final results are true and reliable. However, there are still the following shortcomings in this study: (1) the number of included articles is limited, and the final results may be slightly different from the real results, and this meta is secondary literature and cannot be corrected for multiple tests and report the adjusted p value; (2) there may be some confounding when analyzing across populations because gene frequencies vary between different populations, heterogeneity in some comparisons was not well resolved despite subgroup analyses. Heterogeneity should be considered when interpreting study results, and future studies should focus on more homogeneous patient populations; (3) inability to control factors such as age, gender, weight, and other potential confounding factors may have an impact on the final result; (4) since this subgroup analysis only involved European populations, the results were only for European populations; (5) Although there may be similarities in the genetic susceptibility to Achilles tendinopathy and ACL rupture, the pathologies are not completely identical.

## Conclusion

This analysis did not find any significant relationship between *VEGFA* gene polymorphisms (rs699947, rs1570360, and rs2010963) and the overall risk of tendon and ligament injuries. However, in the European population, individuals with the CC genotype of *VEGFA* rs699947 have a higher risk of experiencing tendon and ligament injuries. Conversely, those with the AG genotype of rs1570360 demonstrate a protective function against these injuries. At the same time, rs2010963 is significant in evaluating the risk of tendon ligament injury. It was statistically determined that individuals carrying the C allele and those with the CC genotype are highly susceptible to this risk. All of the results were validated by FPRP.

## Data Availability

The datasets used and/or analyzed during the current study are available from the corresponding author on reasonable request.
